# Msi1 inhibits cervical cancer cell apoptosis by downregulating BAK through AKT signaling

**DOI:** 10.7150/jca.52950

**Published:** 2021-03-01

**Authors:** Xian Liu, Yanru Zhang, PengSheng Zheng, Nan Cui

**Affiliations:** 1 Department of Reproductive Medicine, The First Affiliated Hospital of Xi'an Jiaotong University, 710061 Xi'an, Shaanxi, PR China.; 2 Section of Cancer Stem Cell Research, Key Laboratory of Environment and Genes Related to Diseases, Ministry of Education of the People's Republic of China, 710061 Xi'an, Shaanxi, PR China.

**Keywords:** Msi1, AKT signaling, apoptosis, cervical cancer

## Abstract

Musashi-1 (Msi1) is an RNA binding protein that functions as a regulator in multiple carcinomas. Our previous study demonstrated that Msi1 could promote the proliferation of cervical cancer cells by targeting the cell cycle proteins P21, P27 and P53. However, the mechanisms by which Msi1 affects the survival of cervical cancer cells, such as apoptosis, are still unclear. In this study, we found that the expression of Msi1 inhibited cervical cancer cell apoptosis *in vitro* and *in vivo*. Furthermore, the expression of Msi1 downregulated the expression of PTEN, while AKT signaling was activated, which resulted in a reduction in the proapoptotic protein BAK. In addition, rescue the expression of BAK in Msi1 expressing cervical cancer cells induced the increase of apoptosis cells. These findings indicate that Msi1 regulates cervical cancer cell apoptosis by inhibiting PTEN and activating AKT signaling, which leads to the downregulation of BAK.

## INTRODUCTION

Cervical carcinoma is a high-incidence malignant tumor that affects the quality of life and threatens the health of females. Human papilloma virus (HPV) is a high-risk factor that accelerates precancerous lesion development to cancer 1. Despite advances in the primary prevention of cervical cancer, a vast majority of patients continue to present at a locally advanced stage, necessitating treatment with chemoradiation and brachytherapy in low- and low-middle-income countries 2. Intractable cervical cancer is still a huge challenge because the median age of patients has dropped and they are suffering from the disease in the middle or late stage, during which treatment is quite difficult. The development of cervical cancer involves a long process of aberrant gene expression and multiple disordered pathways. For example, Slug and SALL4 are related to the progression of cervical cancer 3, 4. ALDH and SOX2 are relevant to cervical cancer stem cells 5,6. Additionally, REX1 is related to the EMT of cervical cancer cells 7 and OCT4 is relevant to cervical cancer cell apoptosis 8.

Msi1, a member of the Musashi family, regulates the expression of proteins by targeting their mRNAs. Msi1 can specifically recognize and bind to the 3'-UTR region of target mRNAs, such as (G/A) UnAGU (n=1-3) 9, 10. Msi1 was first reported to be expressed in neural progenitor cells and during embryonic development. Later, several studies showed that the expression of Msi1 was upregulated in a variety of malignancies, such as glioma, cervical cancer, colorectal cancer, and breast cancer 11, 12, 13, 14. Knockdown of Msi1 could reduce the activities of the PI3 kinase-AKT signaling pathway through the upregulation of PTEN in glioma cells 15. Msi1 activated Notch signaling not only by translationally inhibiting NUMB, but also by downregulating the 26S proteasome by binding to the mRNA of NF-YA in cancer stem cells (CSCs) or tumor-initiating cells 16. Knockdown of Msi1 inactivated STAT3 signaling and inhibited the proliferation of oral squamous cell carcinoma 17. Msi1 could participate in Hedgehog signaling by regulating c-Myc and two microRNAs to regulate the proliferation and apoptosis of mesenchymal stem cells 18. Msi1-associated genes are involved in cell proliferation, cell differentiation, cell cycle, apoptosis and protein modifications 19. It has been reported that Msi1 could promote the EMT, invasion and metastasis of cervical cancer cells by activating the Wnt signaling pathway 20.

A key point for cervical cancer progression is the immortalization of cancer cells. The survival of cervical cancer cells is closely related to proliferation, apoptosis, autophagy, etc. In our previous study, Msi1 accelerated the G0/G1-S cell cycle transformation of cervical cancer cells to promote proliferation by inhibiting the expression of P21, P27 and P53 21. However, the effect of Msi1 on apoptosis in cervical cancer is still unclear. Therefore, the effect of Msi1 on cervical cancer cell apoptosis should be explored *in vivo* and *in vitro*, which might provide a strategy to repress the expression of Msi1 for the treatment of cervical cancer.

## MATERIALS AND METHODS

### Cell culture and clinical samples

The human cervical cancer cell lines SiHa and HeLa were obtained from the ATCC (American Type Culture Collection). Both SiHa and HeLa cells were cultured in high-glucose Dulbecco's modified Eagle's medium (DMEM, Sigma-Aldrich, St Louis, MO, USA). All of the culture media contained 10% FBS (fetal bovine serum, HyClone, Thermo Scientific, Waltham, MA, USA). Bz 423(CAS No:216691-95-1, MedChemExpress, USA), as the agonist of BAK, was added in the culture medium and incubated for 3 hours for BAK rescue experiment. Clinical samples were collected from the First Affiliated Hospital of Xi'an Jiaotong University from 2008 to 2018 as described in our previous study.

### Immunohistochemistry and Immunocytochemistry

The immunohistochemical and immunocytochemical analyses used in this study were performed as described in our previous study. In brief, 4μm sections were prepared from paraffin-embedded tissues, and the tissue sections were subjected to the following process: dewaxing, rehydration, citrate buffer (10 mM sodium citrate, 2 mM citric acid, pH 6.0) incubation for 2 min, 3% H_2_O_2_ for 10 min (room temperature), and PBS washes (phosphate-buffered saline, at 5 min 3 times). Then, the sections were incubated with the primary antibodies at 4°C overnight. The next day, the sections were incubated with secondary antibodies (horseradish peroxidase-conjugated) for 20 mins and washed with PBS 3 times for 5 mins. Finally, the sections were examined under an Olympus CX31 microscope (Olympus, Tokyo, Japan). The sections were scored by 2 individuals in 5 randomly selected representative fields at 40× magnification. The staining intensity was determined as follows: 1, no staining; 2, weak staining; and 3, strong staining. The percentage of positive cells was determined as follows: 1, 0-25% positive cells; 2, 26-50%; 3, 51-75%; and 4, 76-100%. The immunohistochemistry (IHC) score of Msi1 and PTEN = percentage score × intensity score.

The primary antibodies used were as follows: anti-Msi1 (1:100, #H00004440-M04, Abnova, Taipei, Taiwan) and anti-PTEN (1:100, H-134, sc-9081, Santa Cruz Biotechnology, Santa Cruz, CA, USA).

### Apoptosis and flow cytometric analysis

A FACS Annexin V assay kit (BD Biosciences, USA) was used to analyze apoptosis *in vitro*. According to the manufacturer's instructions, 1×10^5^ cells per tube were harvested and stained in triplicate with 5 ml of APC-Annexin V conjugate and 5 ml of propidium iodide (10 mg/ml) for 30 min in the dark. Afterwards, the samples were analyzed by FACSCalibur flow cytometer (Becton Dickinson, Franklin Lakes, NJ, USA) with CellQuest software. The apoptotic index was calculated as the percent of apoptotic cells.

### TUNEL assay

The preparation of xenograft tumors was described in our previous study 21. Tissue slides were prepared from the xenograft tumors by paraffin embedding. According to the manufacturer's instructions, TUNEL staining was performed by the DEAD END colorimetric TUNEL system (Promega). Apoptotic cells were analyzed by counting the total amount of TUNEL-positive nuclei in 10 random fields and excluding cells undergoing mitosis.

### Western blotting

Western blotting was performed as previously described 21.

The antibodies used were as follows: anti-Msi1 (1:1000 dilution, Abnova, Taipei, Taiwan), anti-PTEN (1:1000 dilution, Santa Cruz, CA, USA), anti-PI3K (1:1000 dilution, Santa Cruz, CA, USA), anti-p-AKT (1:1000 dilution, Cell Signaling Technology), anti-AKT1 (1:1000 dilution; Santa Cruz, CA, USA), anti-mTOR (1:1000 dilution, Santa Cruz, CA, USA), anti-BAK (1:500 dilution, Santa Cruz Biotechnology), anti-Bcl-2(1:1000 dilution, Santa Cruz, CA, USA), anti-GAPDH (1:1000 dilution, Santa Cruz, CA, USA), and the secondary antibody coupled to horseradish peroxidase (Goat aiti-Mouse:1:10000 dilution; Goat anti-Rabbit: 1:15000, Thermo Fisher Scientific Inc., New York, NY, USA). The proteins were visualized with an enhanced chemiluminescence reagents (Millipore, Billerica, MA, USA) with the protein imprinting imaging system (Tanon 5200, China). GAPDH was used as the control and for quantification.

### Statistical analysis

Statistical Package of Social Science (SPSS) software version 18.0 (SPSS Inc., Chicago, IL, USA) was used for statistical analyses. Univariate analyses were performed using Student's t-tests or one-way ANOVA. The correlations among expression levels of different genes were determined by using the Pearson correlation analysis. In all of the tests, statistical significance was defined as *P*<0.05.

## RESULTS

### Msi1 enhances the survival of cervical cancer cells

**Figure 1:**
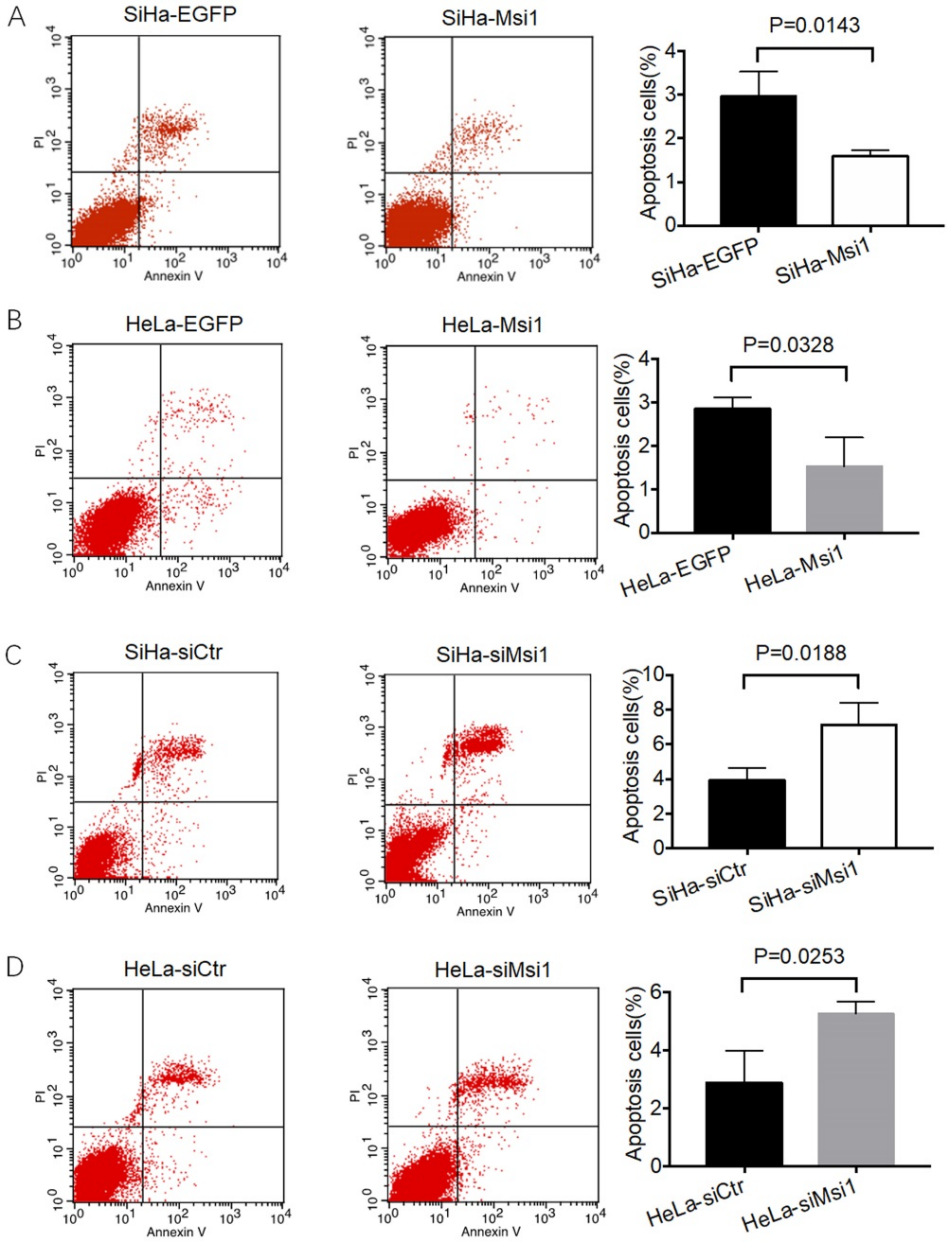
** Msi1 inhibited cervical cancer cell apoptosis *in vitro*.** Flow cytometric apoptosis analysis was performed on control cervical cancer cells and Msi1-modified cervical cancer cells. (A) The percentage of apoptotic cells in the SiHa-EGFP group was 2.973±0.322% and that in SiHa-Msi1 group was 1.597±0.081% (Student's t-test, P=0.0143); (B) The percentage of apoptotic cells in the HeLa-EGFP group was 2.847±0.153% and that in the HeLa-Msi1 group was 1.523±0.384% (Student's t-test, P=0.0328); (C) The percentage of apoptotic cells in the SiHa-siCtr group was 3.910±0.410% and that in the SiHa-siMsi1 group was 7.113±0.732% (Student's t-test, P=0.0188); (D) The percentage of apoptotic cells in the HeLa-siCtr group was 2.897±0.637% and that in the HeLa-siMsi1 group was 5.263±0.237% (Student's t-test; P=0.0253).

As our previous study showed, Msi1-overexpressing SiHa and HeLa cells, as well as siMsi1-expressing SiHa and HeLa cells, were identified. Msi1 promotes the proliferation of cervical cancer cells both *in vitro* and *in vivo* by inhibiting the expression of P21, P27 and P53, which are key regulators of the G0/G1 phase to S phase transition in the cell cycle 21. Furthermore, a flow cytometry-based apoptosis assay was also performed. As shown in Figure 1A and 1B, the percentages of apoptotic Msi1-overexpressing SiHa and HeLa cells were significantly lower than those of their respective control cells (SiHa-Msi1 vs SiHa-EGFP: *P*<0.05, Student's t-test; HeLa-Msi1 vs HeLa-EGFP: *P*<0.05, Student's t-test). In contrast, SiHa- and HeLa-siMsi1 cells exhibited higher percentages of apoptotic cells than their respective controls, which are shown in Figure 1C and 1D (SiHa-siMsi1 vs. SiHa-siCtr: *P*<0.05, Student's t-test; HeLa-siMsi1 vs HeLa-siCtr: *P*<0.05, Student's t-test). These results indicated that Msi1 maintained the survival of cervical cancer cells by not only promoting their proliferation but also inhibiting apoptosis in the cancer cells.

### Msi1 inhibits cervical cancer cell apoptosis *in vivo*

**Figure 2:**
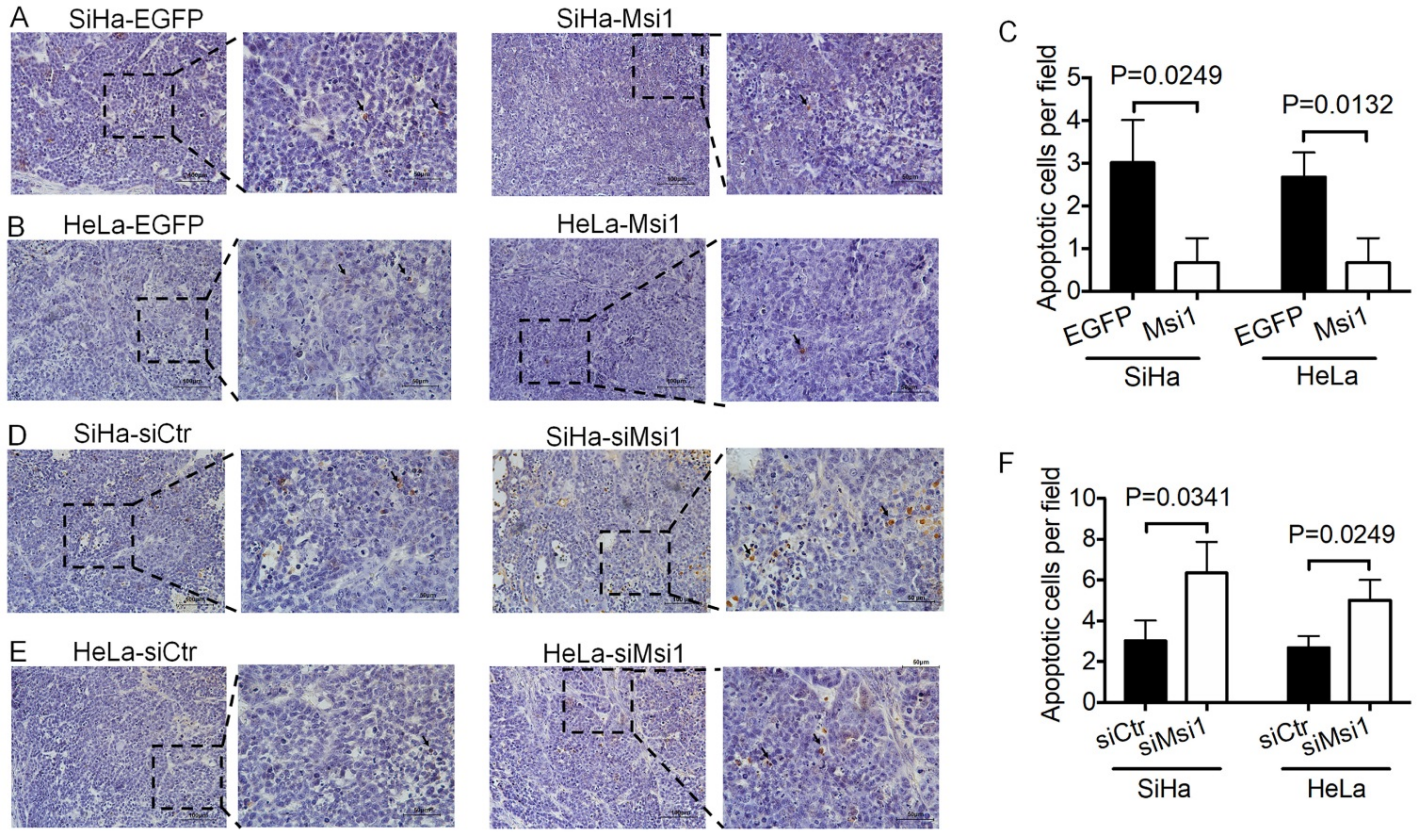
** Exogenous Msi1 inhibited cervical cancer cell apoptosis *in vivo*.** TUNEL assays were performed in triplicate on mouse xenograft tumors from the SiHa-EGFP and SiHa-Msi1 (A), HeLa-EGFP and HeLa-Msi1 (B), SiHa-siCtr and SiHa-siMsi1(D), HeLa-siCtr and HeLa-siMsi1 groups (E). Brown colored cells were pointed out as TUNEL^+^ cells. Scale bar= 100 μm for the main image and 50 μm for local image of TUNEL^+^ cells. (C) and (F), the data are presented as the mean±S.E.M. using Student's t-tests, and the respective *P* values are marked.

In our previous study, xenografted tumors formed by Msi1-modified cells were described 21. Furthermore, tissue sections of xenografted tumors were used for TUNEL assays. Tumors formed by Msi1-expressing cells exhibited decreased numbers of apoptotic cells compared with those of the control groups (Figure 2A-2C, SiHa-Msi1 vs SiHa-EGFP: *P<0.05*, Student's t-test; HeLa-Msi1 vs HeLa-EGFP: *P<0.05*, Student's t-test). In contrast, an increase in the number of apoptotic cells in tumors formed by siMsi1 cells was determined by TUNEL assays, as shown in Figure 2D-2F (SiHa-siMsi1 vs SiHa-siCtr: *P<0.05*, Student's t-test; HeLa-siMsi1 vs HeLa-siCtr: *P<0.05*, Student's t-test). These results indicated that Msi1 promoted tumor formation by inhibiting apoptosis in cervical cancer tissue.

### Msi1 activates PI3K/AKT signaling and downregulates PTEN and BAK

**Figure 3:**
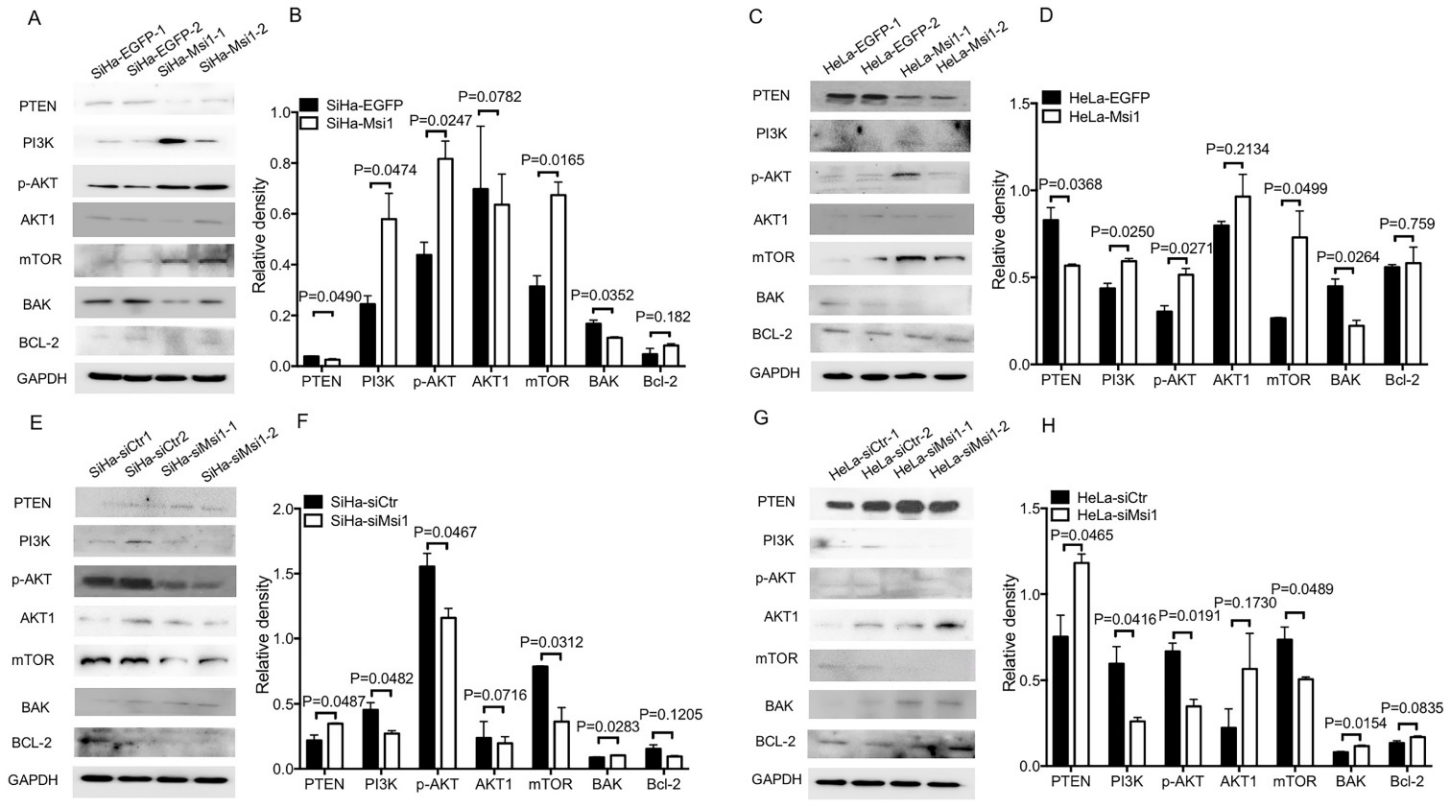
** Downregulation of PTEN expression, activation of AKT signaling and downregulation of BAK expression by Msi1.** (A) The expression levels of PTEN, PI3K, p-AKT, AKT1, mTOR, BAK, and Bcl-2 in SiHa-EGFP and SiHa-Msi1 cells were measured by western blotting, and the quantitative analysis is shown (B). (C) The expression levels of PTEN, PI3K, p-AKT, AKT1, mTOR, BAK, and Bcl-2 in HeLa-EGFP and HeLa-Msi1 cells were measured by western blotting, and the quantitative analysis is shown (D). (E) The expression levels of PTEN, PI3K, p-AKT, AKT1, mTOR, BAK, and Bcl-2 in SiHa-siCtr and SiHa-siMsi1 cells were measured by western blotting, and the quantitative analysis is shown (F). (G) The expression levels of PTEN, PI3K, p-AKT, AKT1, mTOR, BAK, and Bcl-2 in HeLa-siCtr and HeLa-siMsi1 cells were measured by western blotting, and the quantitative analysis is shown (H). GAPDH was used as the internal reference. The densitometric analysis data are shown as the means ± S.D. of three independent experiments using one-way ANOVA, and the *P* values are marked.

PI3K/AKT signaling has been suggested to play an important role in the proliferation, apoptosis and migration of tumors. Previous studies have shown that Msi1 can activate AKT signaling in lung cancer and glioblastoma to promote malignancy 22,23. Thus, key members of the AKT signaling pathway were examined by western blotting and densitometry analysis. As shown in Figure 3A-3D, the expression levels of PI3K and p-AKT in Msi1-overexpressing cells were upregulated (PI3K, *P<0.05*; p-AKT, *P<0.05*, one-way ANOVA), while the expression of PTEN, a negative regulator of AKT signaling, was downregulated (*P<0.05*, one-way ANOVA). Moreover, BAK, which is downstream of AKT signaling and functions as an apoptotic factor, was downregulated (*P<0.05*, one-way ANOVA). In contrast, as shown in Figure 3E-3H, in siMsi1 cells, the expression levels of PI3K and p-AKT were downregulated (PI3K, *P<0.05*; p-AKT, *P<0.05*, one-way ANOVA), the expression of PTEN was upregulated (*P<0.05*, one-way ANOVA), and BAK was upregulated (*P<0.05*, one-way ANOVA). These results suggested that Msi1 inhibited cervical cancer cell apoptosis through AKT signaling by inhibiting PTEN. Notably, the expression of mTOR was increased in Msi1-overexpressing cells but decreased in siMsi1-modified cells, suggesting another possible mechanism by which Msi1 promotes the proliferation of cervical cancer cells (Figure 3A-3H).

### PTEN and BAK inversely correlate with Msi1 expression

**Figure 4:**
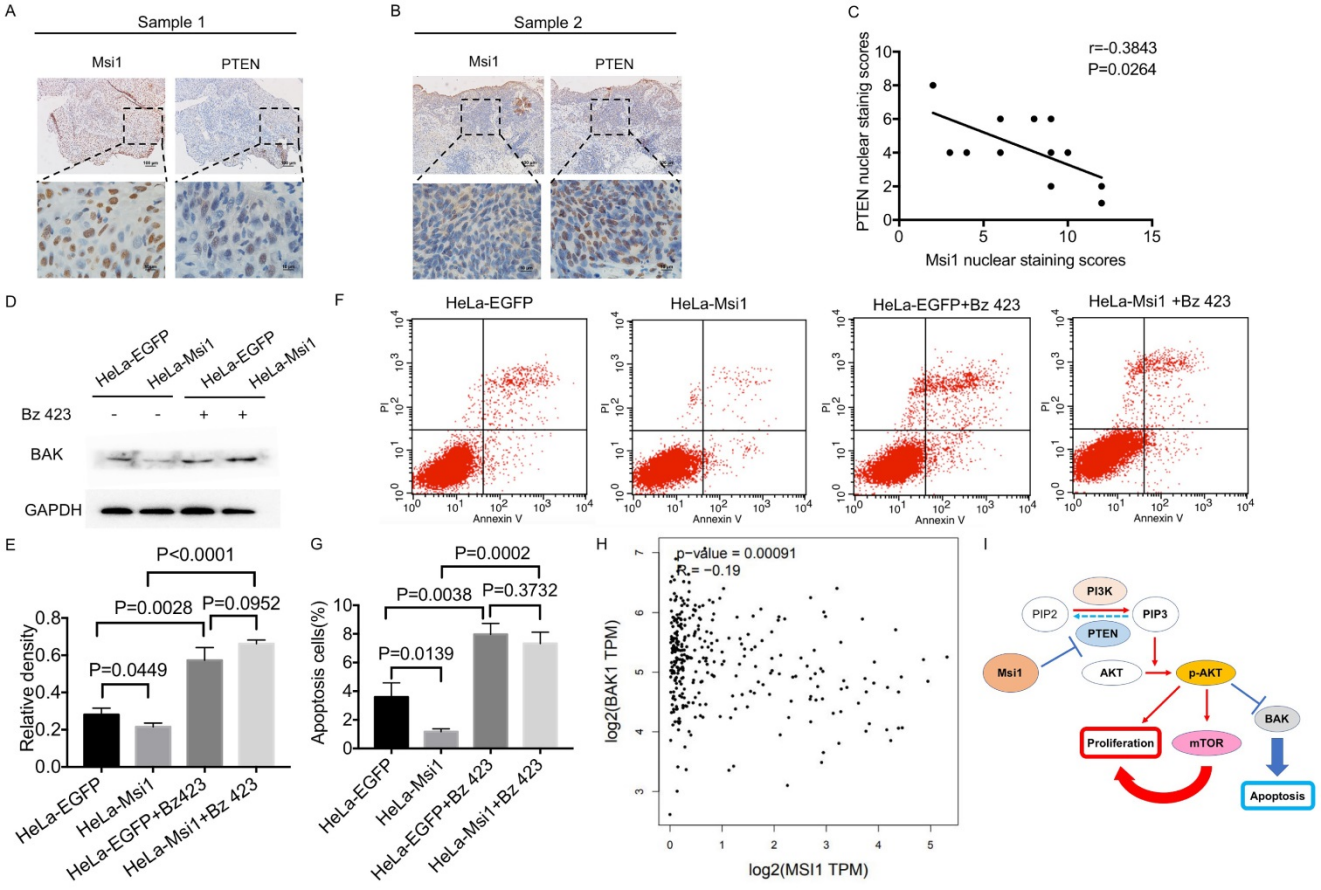
** Msi1 negatively correlated with PTEN and BAK in cervical cancer.** The expression levels of Msi1 and PTEN in two representative samples are shown (A) and (B). Scale bars= 100μm for upper panel, 10μm for lower panel. (C) PTEN nuclear staining scores and respective Msi1 nuclear scores were scattered with the correlation ratio and *P* value (r=-0.3843, *P*=0.0264). (D) and (E), western blot and relative density analysis of BAK in HeLa-EGFP cells and HeLa-Msi1 cells with or without treatment of 10μM Bz 423. (F) and (G), flow cytometric apoptosis analysis was performed in HeLa-EGFP cells and HeLa-Msi1 cells with or without treatment of 10μM Bz 423. The percentage of apoptotic cells in HeLa-EGFP cells was 3.593±0.568% and that in HeLa-Msi1 cells was 1.167±0.120%. With the treatment of Bz 423, the percentage of apoptotic cells in HeLa-EGFP cells was 7.960±0.461% and that in HeLa-Msi1 cells was 7.320±0.457%. *P* values were marked in the figure. (H) The correlation analysis of Msi1 and BAK from data of GEPIA which sourced from cervical cancer samples and normal cervix samples. R value and *P* value was marked. (I) Schematic representation of the mechanism by which Msi1 activates AKT signaling and thereby inhibiting apoptosis of cervical cancer cells.

In 12 cervical cancer samples, Msi1nuclear staining score was 7.50±3.31 and PTEN nuclear staining score was 4.25±2.00. The scatterplot of PTEN and Msi1 correlation was shown in Figure 4C. The levels of PTEN were negatively correlated with the expression of Msi1 in these clinical samples, indicating that PTEN downregulation occurred in human cervical carcinoma tissues (Figure 4A-4C*,* r=-0.3843, *P*=*0.0264*). These results suggest that Msi1 might promote cervical cancer development by inhibiting apoptosis via PTEN. To confirmed the critical role of BAK in Msi1-PTEN-AKT pathway, rescue experiment was performed. Bz 423(10μM), an activating agent of BAK, was used to exert the expression of BAK in HeLa Msi1-overexpressing cells and respective control cells. As shown by western blot in Figure 4D and 4E, the expression of BAK were significantly rescued by Bz 423 in both HeLa-Msi1 cells and HeLa-EGFP cells (HeLa-EGFP+Bz423 vs HeLa-EGFP: *P<0.05*; HeLa-Msi1+Bz423 vs HeLa-Msi1: *P<0.05*, one-way ANOVA). The apoptosis of cervical cells was significantly increased in HeLa-Msi1 cells treated with Bz 423 compared with that in HeLa-Msi1 cells without Bz 423 treatment by the flow cytometry-based apoptosis assay (Figure 4F and G; HeLa-Msi1+Bz423 vs HeLa-Msi1: *P<0.05*, Student's t-test). These results suggested that BAK might play a functional role of apoptosis in Msi1-PTEN-AKT signaling. Furthermore, the analysis of cervical cancer samples and normal cervix samples data from GEPIA (gepia.cancer-pku.cn) also present a negative correlation between Msi1 and BAK (Figure 4H; r=-0.19, *P=0.0009*).

## DISCUSSION

The occurrence and development of cervical cancer is a relatively long process. Although HPV infection is an important cancer-promoting factor, the abnormal expression of multiple tumor suppressors and promotors in the complex internal environment of the body are important factors for the continuous progression and worsening of cervical cancer. The survival of cancer cells depends on the proliferation of cells as well as the death of cells. Apoptosis is a classic form of cell death. Previous studies have shown that Msi1 can promote the proliferation of a variety of tumors cells 24. Moreover, silencing of Msi1 induces apoptosis in esophageal squamous cell carcinoma and bladder carcinoma cells, while knockdown of Msi-1 by small interfering RNA (siRNA) promotes apoptosis in ovarian carcinoma 25, 26, 27. Consistently, in our study, cervical cancer cells exhibited resistance to apoptosis in response to exogenous expression of Msi1 both *in vitro* and *in vivo* (Figures 1 and 2).

Activation of the AKT pathway can regulate cell survival, proliferation and glucose metabolism 28. PTEN can inactivate AKT signaling and block downstream AKT signaling by dephosphorylating PIP3 29. It was reported that a decrease in Msi1 expression could upregulate PTEN expression and inhibit AKT signaling activity in glioma 15. Msi1 bind and regulate mRNA stability and translation of proteins operating in essential oncogenic signaling pathway including PTEN/mTOR 24. As shown in our study, PTEN expression was upregulated in the presence of Msi1 expression, followed by activated AKT signaling, which was similar to the results of the abovementioned study. In addition, the expression of PTEN was downregulated in the presence of exogenous expression of Msi1 that facilitated the expression of PI3K and p-AKT. These results indicated that Msi1 expression could activate AKT signaling. On the other hand, BAK is downstream of AKT signaling and participates in cancer cell apoptosis 30. mTOR, including mTOR1 and mTOR2, is another downstream target of AKT signaling that is mainly associated with proliferation, autophagy and the regulation of AKT signaling 31,32. As shown in Figure 3, overexpression of Msi1 impaired the expression of BAK, and inhibiting Msi1 expression increased its expression in cervical cancer cells. In addition, Msi1 increased the expression of mTOR, suggesting that Msi1 accelerated the proliferation of cervical cancer cells might not only through regulating the cell cycle, but also modulating the AKT signaling. Notably, Msi1 might participate in the autophagy process, which remains unclear. Furthermore, AKT signaling inactivates the tumor suppressor gene TP53, which drives cancer cells to proliferate and escape preprogrammed cell death 33. While we proved that Msi1 could regulate P53 directly by binding to its 3'-UTR, whether Msi1 regulates apoptosis indirectly through the AKT/P53 pathway remains to be confirmed.

The correlation between Msi1 and PTEN in clinical samples was determined in our study, which suggested that Msi1 negatively regulated the expression of PTEN (Figure 4). Potential binding sites of Msi1 in the PTEN 3'-UTR were screened, and there were a total of 18 regions containing (G/A)U_1-3_AGU. It had been confirmed that Msi1 could bind to the 3' UTR of the PTEN mRNA, decreasing PTEN protein levels in human colorectal cancer (CRC) cells 34. However, whether Msi1 binds to the motif in the 3'-UTR containing the above sequence still needs to be confirmed in cervical cancer cells. Furthermore, BAK was downregulated in Msi1 expressing cells, and was upregulated in rescue experiment of BAK in these cells, which lead to the increase of the number apoptosis cells. Correlation of Msi1 and BAK also exhibited a negative tendency analyzed by GEPIA (Figure 4). However, whether this effect is direct or indirect will be verified in our subsequent works.
